# Ginger Essential Oil as an Active Addition to Composite Chitosan Films: Development and Characterization

**DOI:** 10.3390/gels8060327

**Published:** 2022-05-24

**Authors:** Sawsan Ali Al-Hilifi, Rawdah Mahmood Al-Ali, Anka Trajkovska Petkoska

**Affiliations:** 1 Department of Food Science, College of Agriculture, University of Basrah, Basrah 61014, Iraq; rawdah.ali@uobasrah.edu.iq; 2 Faculty of Technology and Technical Social Sciences, St. Kliment Ohridski University-Bitola, Dimitar Vlahov, 1400 Veles, North Macedonia; anka.trajkovska@uklo.edu.mk

**Keywords:** antimicrobial packaging, ginger essential oil, chitosan films, biodegradable materials

## Abstract

The recent interest in food biopackaging is showing an increasing trend, especially in the development of antimicrobial coatings and films. The focus of this study is to assess the potential application of ginger (*Zingiber officinale)* essential oil (GEO) to polysaccharide films based on chitosan (CHf) and their utilization as an active edible packaging. The films were characterized by different instrumental techniques, and data indicated significant differences (*p* < 0.05) in the chemical composition of the samples. Forty-seven active compounds from ginger rhizomes were identified in the examined essential oil by gas chromatography mass spectrometer (GC-MS). Fourier transforms infrared spectra (FT-IR) confirmed an interaction between the hydroxyl groups of the phenolic compounds of the essential oil and the amine groups of the bioactive matrix, as shown by the peaks at wavenumbers 1639 cm^−1^ and 1558 cm^−1^. X-ray diffraction data suggested a lower crystallinity in the CHf due to the addition of GEO. Differential scanning calorimetric (DSC) analysis revealed that the CHf possessed high thermal stability, especially when different concentrations of GEO were added. The bioactive CHf showed distinct activity against both Gram-positive and Gram-negative bacteria, such as *Staphylococcus aureus*, *Bacillus subtilis*, *Streptococcus* sp., *Escherichia coli*, *Salmonella* sp., and *Pseudomonas aeruginosa*, thus improving the antimicrobial activity to these films. The results provide a comprehensive insight into the importance of films with incorporated EOs as novel types of active food packaging. Antimicrobial food packaging is one of the most promising kinds of active packaging, and acts to reduce, inhibit, or retard any microorganism growth that could contaminate packaged food items.

## 1. Introduction

Active packaging (AP) has an attractive option in the food industry over the last few decades. Natural antimicrobial materials combined with edible materials can prevent or control outbreaks of infectious foodborne pathogens, extend the shelf-life of food products and improve the quality and safety standards of the packaged items. The demand for biodegradable and eco-friendly packaging is expected to reach high levels in the food packaging market around the world [1,2,3]. Biopolymers, such as proteins, polysaccharides, and lipids, meet the requirements for biodegradable and edible films and coatings. They can be used individually, or combined with other biopolymers or mixed with other compounds in order to obtain edible films or coatings suitable for food packaging. These films/coatings can cover the entire surface of the food and protect it from mechanical damage and light/UV exposure, serve as a gas/water vapor barrier, and generally improve the quality and safety of packed food products [4,5]. In general, AP represents a transformation of the role of the passive packaging system to one of active defense. It catalyzes the interaction between the environment, packaging, and the product for longer shelf life and/or better quality of packed food. It involves the intentional combination of the absorption or discharging of certain compounds from or into the food packaging system [6,7]. These active systems usually contain active substances that can be released into the packaging interior [8,9,10]. Moreover, biopolymer-based films have proven to be an exceptional matrix for developing functional (active) packaging materials with the inclusion of a variety of additives, such as antioxidants and antimicrobial agents. As they inhibit the spread of pathogenic microbes that cause food spoilage and contamination [11,12], active films can play an important role in packaging and preservation of fresh food, such as fruits, vegetables, dairy products, meat, and meat products [13,14,15,16]. In this context, biocomposite polysaccharide materials are gaining significant attention due to the abundant availability of such biopolymers and their proven track records in various applications [17,18,19,20].

Essential oils (EOs) with antimicrobial attributes present a promising bioactive addition to packaging technology. From a chemical point of view, EOs are composed of secondary metabolites, the majority of which are terpenes (especially monoterpene) containing oxygenated derivatives and other compounds, such as hydrocarbons, ethers, alcohols, aliphatic acid esters, and phenolics, which are responsible for antibacterial activity [21,22,23]. The antimicrobial and antioxidant properties of EOs are numerous, and they can be employed as a substitute for synthetic preservatives in the food industry [24]. Furthermore, EOs can be incorporated into biopolymer materials, such as chitosan, to enhance the antimicrobial effect, and they have Generally Recognized as Safe (GRAS) status [25,26]. Essential oil of ginger (*Zingiber officinale*), abbreviated here as GEO, is well recognized for its antimicrobial and oxidative actions [27], which are usually attributed to active compounds such as zingiberene, camphene, α-curcumene, and α-phellandrene. In addition, EOs contain mixtures of chemical compounds, such as hydrocarbons and oxygenated monoterpene [28,29]. Chitosan (CH) is a homogeneous polymer of *N*-acetyl-d-Glucose units linked by β-(1→4) bounds, and can be found in the exoskeletons of crustaceans. CH is a natural, sustainable and biocompatible material with exciting and unique antimicrobial properties, and is used in the pharmaceutical industry, for food packaging, and in environmental conservation [30,31,32]. The activity of CH is due to its reactive amino and hydroxyl functional groups, but it is frequently blended with other polymers [33]. The resistance of bacteria and fungi to chemicals is a major challenge in the treatment of infections, leading to the need for new materials with anti-bacterial properties. The combination of essential oils and chitosan films coating may enhance such materials [34]. The aim of this study is to highlight the effectiveness of antimicrobial chitosan films (CHf) as well as to study their chemical properties. In this work, the authors focus on the development of active biopackaging films using GEO combined with CHf, assessing the effect of this combination on the thermal stability. properties of the produced films. Moreover, the efficiency of GEO-enriched films in inhibiting the growth of both Gram-positive and Gram-negative foodborne pathogenic bacteria is also determined.

## 2. Results and Discussion

### 2.1. Chemical Components of GEO

**Figure 1:**
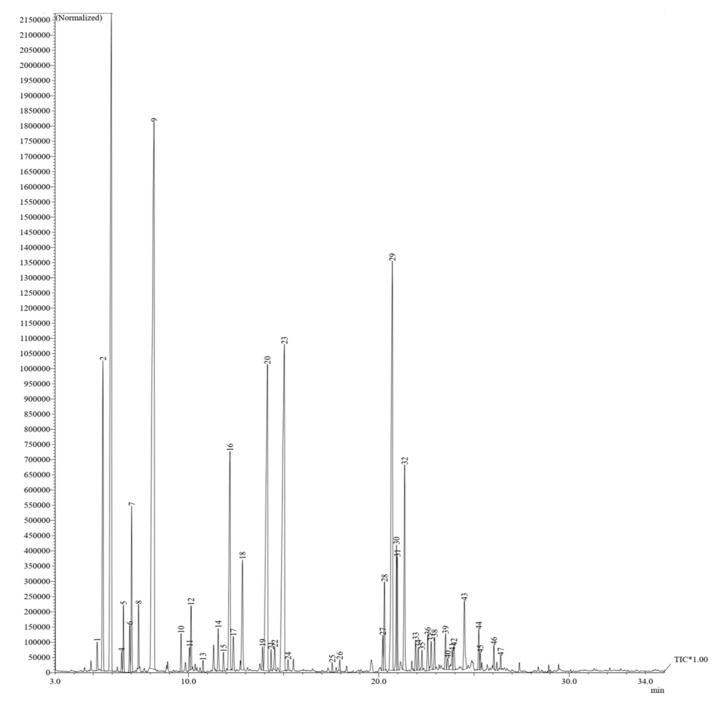
Typical chromatogram of total ion current plot of essential oil isolated from the rhizomes of ginger obtained by GC\GC–MS analysis.

GC-MS analysis of GEO showed a composition of different components (Table 1). Oxygenated monoterpenes, monoterpene hydrocarbons, diterpene hydrocarbons, sesquiterpene hydrocarbons, oxygenated sesquiterpenes, diterpene hydrocarbons, and fatty acid esters were all present in this EO. The most essential components were eucalyptol (19.36%), (−)−camphene (15.07%), β-bisabolene (11.52%), zingiberene (9.58%), and cineol (9.18%) (Figure 1). Several compounds found in GEO, such as (−)−camphenein, alpha-curcumen, and α-zingiberene, have known antibacterial properties. Moreover, the antibacterial properties may also be attributed to α-phellandrene, α-pinene, β-selinenol, β-sesquiphellandrene, limonene, and linalool [25,35]. The major chemical constituents of GEO found in the current study were similar to those found in previous studies [36]. The minor variations in concentrations are due to the changes in internal and external factors related to the growth environment, harvest season, and extraction process used. Wang et al. (2020) [25] found that β-phellandrene, α-curcumene, and α-zingiberene in the GEO were concentrationof 2.56%, 12.04%, and 35.65%, respectively [37]. α-Zingiberene has also been identified by Teerarak & Laosinwattana (2019) [38] as one of the most abundant components of this oil.

### 2.2. Chemical Composition of Chitosan

The chemical compounds of fresh shrimp shell and the CH synthesized from it are shown in Table 2. The content of moisture, protein, and ash in the shrimp shell were 44.25%, 33.57%, and 31.40%, respectively, whereas the values of protein and ash (3.72% and 1.48%) decreases in prepared chitosan compared to raw shells. The ash percentage is directly related the calcium carbonate in the shrimp shell [39]. These results indicate the efficiency of the demineralization, which is an essential step in preparing chitosan, as this treatment leads to the removal of the largest amount of calcium carbonate and calcium phosphate, whose concentration in the shell is about 30–50% [40]. These results are similar to the study conducted by Samar et al. (2013) [41] who found moisture, protein and ash contents of 45.65%, 32.45%, and 32.77%, respectively [42].

### 2.3. Fourier Transform Infrared Spectroscopy (FT-IR)

**Figure 2:**
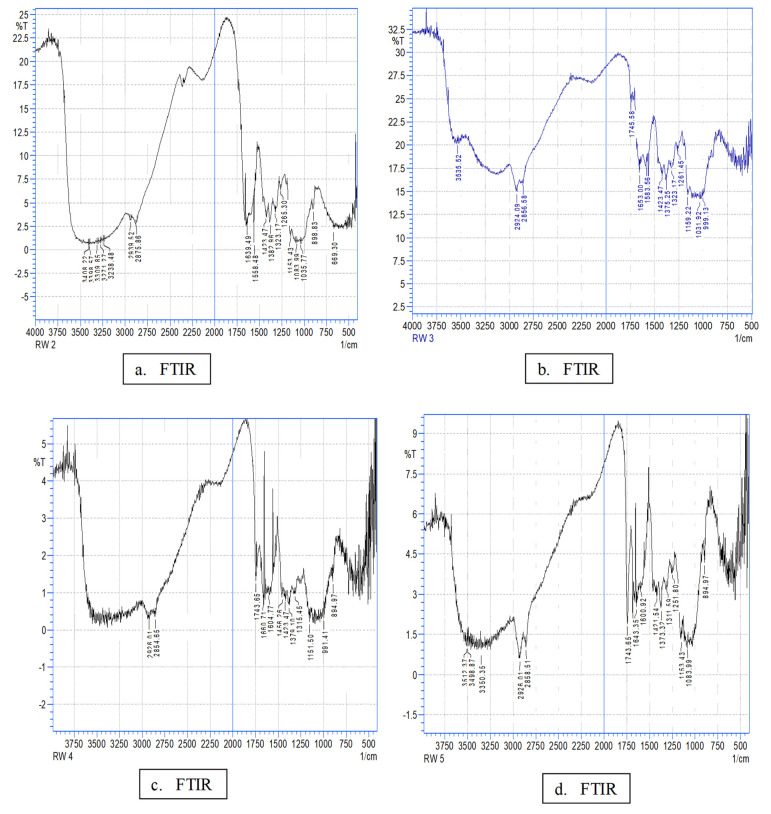
FT-IR spectrum of chitosan films with incorporated GEO at different concentrations. (**a**) Chitosan; (**b**) Chitosan + 0.1% GEO; (**c**) Chitosan + 0.2% GEO; (**d**) Chitosan + 0.3% GEO.

FT-IR analysis of the films was used to ascertain the changes generated by the incorporation of GEO into biofilm interactions by separating the IR bands and vibrational shifts related to the incorporation of GEO into the film. The spectrum of bioactive films (Figure 2) revealed distinct bands at around 3500 cm^−1^ to 3000 cm^−1^ (NH bond), at 1630–1400 cm^−1^ (C=O bond) [43,44], at 3398 cm^−1^ (axial stretch of –OH), and at 3271 cm^−^^1^ (an asymmetric extension of –NH group). The wide band in the range 3400–3000 cm^−1^ is attributable to the O–H and N–H stretching vibration. The band in the range 2939 cm^−1^ to 2875 cm^−1^ is ascribed to the C–H bond to –NHCOCH_3_ of the methyl group; the band at 1639 cm^−1^ indicates amide I and C=O stretching; the band at 1558 cm^−1^ is attributable to amide II and N–H bending; the band in the range 1423–1382 cm^−1^ indicates CH_2_ bending; the band in the range 1083 cm^−1^ to 898 cm^−1^ is attributable to skeletal vibration, including the stretching of the C–O group, and the band in the range 1155 cm^−1^ to 1265 cm^−1^ to asymmetric stretching of the C–O–C [45,46,47,48]. In general, the incorporation of GEO modulation resulted in slight differences in spectra compared to the control CHf; this may have been due to the small amount of GEO incorporated, CH characteristic peaks prevailed in all samples. Slight changes in absorption peaks were recorded due to the overlapping of chemical bonds, and thus are considered evidence of an interaction between the molecules of different components. The FT-IR spectra of the GEO composition films show a new peak between 1745 cm^−1^ to 1743 cm^–1^ that corresponds to the vibration of the C=O bond stretch; this represents an interaction between the hydroxyl and amine groups from CH with the phenolic compounds from the GEO [49].

### 2.4. Thermal Properties of Chitosan Films

**Figure 3:**
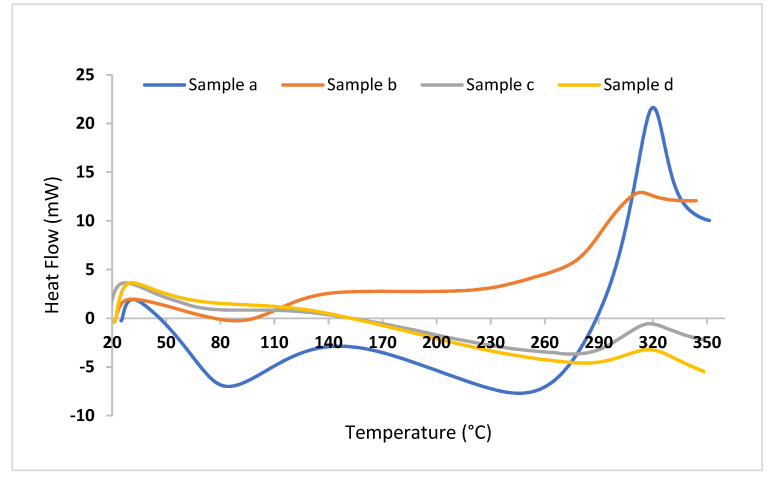
Differential scanning calorimetry of tested films: (**a**) Chitosan; (**b**) Chitosan + 0.1% GEO; (**c**) Chitosan + 0.2% GEO; (**d**) Chitosan + 0.3% GEO.

The thermal analysis method is one of the physical methods for evaluating a polymer’s endurance and stability within temperature range. In this study, DSC was used to estimate the tolerances of CH and GEO films, as presented in Figure 3 and Table 3. The results show that the CHf recorded the lowest melting point (Tm) at 88.51 °C and endothermic peak at 314.03 °C, which may be attributed to the moisture loss associated with the hydrophilic groups in chitosan and polymer decomposition that accounts for its crystallinity [50]. It is notable that the highest value of the enthalpy of melting (∆H) in the CH/0.3% GEO is 526.02 J/g and the lowest in the CH is 69.01 J/g. The greater the value of the change in the enthalpy in the model, the greater its ability to withstand high temperatures (Figure 3). In other words, the change in the model’s entropy value increases with the increase in the degree of crystallization, and therefore, the more highly crystallized model is more resistant to higher temperatures. Increased crystallinity results in higher elasticity and in a greater tendency toward breakage (lower TS) [51]. It is evident from the results that there is a direct relationship between the oil concentration in the films and crystallization; this may be caused by the movement of CH molecules into polymer segments, thus making it easier to arrange the polymer chain [52].

### 2.5. X-ray Diffraction (XRD)

**Figure 4:**
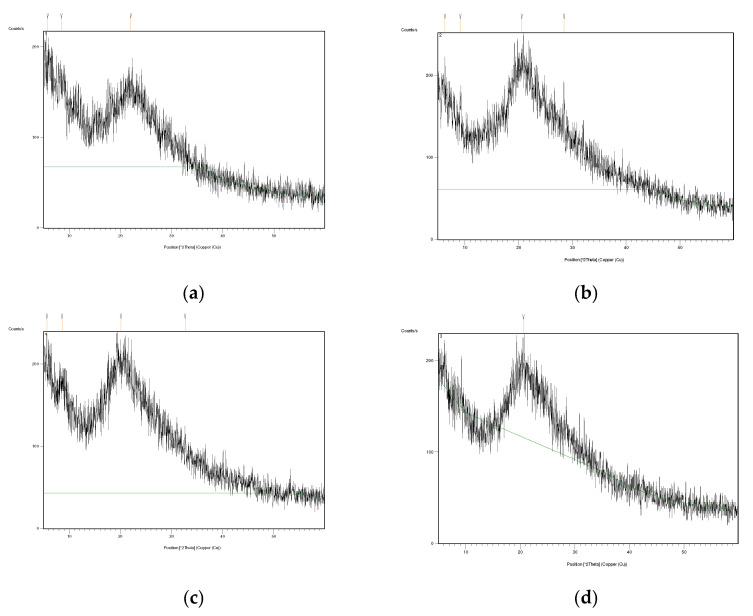
X-ray Diffraction patterns of tested films: (**a**) Chitosan; (**b**) Chitosan + 0.1% GEO; (**c**) Chitosan + 0.2% GEO; (**d**) Chitosan + 0.3% GEO.

X-ray diffraction analysis is used to ascertain the crystal structure of a substance by determining the internal atomic space. XRD patterns of CHf with different amounts of GEO are shown in Figure 4. The blunt peaks at 2θ (ranging between 5–20) are in agreement with the assumption that CH is a molecularly crystallized polysaccharide that contains some crystals embedded in the amorphous region [53]. CH has the advantage of being able to take three forms: hydrated crystalline, anhydrous crystalline and non-crystalline [54]. The control CHf appeared in a crystalline state with a major diffraction peak at 5.7° (2θ). These results were consistent and in agreement with Sun et al. (2017) [55] with a diffraction peak at 8.44°. According to a previous study [56], the diffraction peak at 11.54° is attributable to the anhydrous crystalline, while at 18.34° the diffraction peak is the aqueous crystal character [57]. Moreover, it has been observed that a diffraction peak at 22.8° is considered a typical fingerprint for CHf [58]. It was observed that the diffraction peaks became flatter and less discernible when adding GEO to the films, indicating a decrease in crystallinity with increasing concentrations of GEO. The crystallinity index values of films are usually lower when they are combined with EOs due to the close packing in the polymer chains; this could be attributed to the stronger interaction between active compounds and biopolymers (i.e., H-bonds, Van der Waals), which leads to an improvement in the crystalline 3D-network [59,60].

### 2.6. Antimicrobial Activity

The antimicrobial activity of chitosan bioactive films containing GEO is presented in Table 4. CHf with 0.3% GEO showed the lowest antimicrobial activity compared to other films that were studied against the tested microorganisms. The mechanism of antimicrobial activity of CH is based on its positive charges (NH^3+^) which interfere with the electro-negatively charged cell membrane. Leakage of proteinaceous, ionic, and other intracellular materials causes cell death [61,62,63,64]. CH cannot disperse through agar media, and only species that come into contact with the active areas of CH are affected [65,66,67]. In this study, the growth of the six pathogens was inhibited by all bioactive films to varying degrees depending on GEO concentration. Zone of inhibition increased significantly (*p* < 0.05) as the concentration of GEO in the films increased. CHf with the highest concentration of GEO (0.3% *w/v*) effectively inhibited the growth of tested bacteria, resulting in halos from 9.02 mm to 18.35 mm. The antibacterial activity of GEO is thought to be due to its high phenolic content [68,69]. In general, GEO has a stronger effect on Gram-positive bacteria than on Gram-negative bacteria [70]; GEO was found to have antibacterial activity against *S. aureus* by Trajano et al. [71], while Singh et al. (2008) [72] found strong antimicrobial activity against *E. coli*, *S. aureus*, and *Yersinia enterocolitica*. This could be the result of synergism generated by the mixture of CH and GEO due to the presence of terpenoids that have antibacterial effects, such as zingerone, shogaol, nerolidol, and other phenolic compounds [73,74]. Analysis of red ginger oil with the GC-MS technique by Irawan et al. (2019) [75] found that it also contains compounds such as curcumene (15.6%), zingiberene (10.3%), β-sesquiphellandrene (8.74%), cineole (7.52%), α-pinene (3.12%), and borneol (0.46%), all of which have antibacterial properties. The effect of GEO on microorganisms is due to several mechanisms, including changes to the permeability of the cell membrane, inhibition of intracellular metabolic pathways, and disruption of enzyme systems, which affects the genetic material of the bacteria, because the aromatic and phenolic compounds of the oils affect the cytoplasmic membrane and change its function [76,77]. This is confirmed by Remya1 et al. (2016) [78] who also showed that addition of GEO improves the antibacterial properties of CHf.

## 3. Conclusions

In this study, the incorporation of GEO into chitosan was successful in the preparation of bioactive films intended for active food packaging. The characterization of biocomposite active films was determined using several methods of analysis. The effect of incorporating GEO into bioactive films based on CHf/GEO was assessed by identifying both the melting temperature (Tm) and an endothermic peak (Tp), which indicated a semi-crystalline structure. The contact angle was reduced by the addition of GEO, and thus it can be concluded that CHf/GEO films have a more hydrophobic surface than pristine CHf. Semi-crystalline polymer crystallographic morphology is noticeably changed by the effect of EO. The molecular interactions that occurred after the addition of GEO were characterized using FT-IR analysis. The films were effective against foodborne pathogens in general. Food packaging is critical for preventing bacterial contamination. Biodegradable food packaging films, such as those described in this work, are promising in the long term because they are composed of environmentally friendly materials. In light of these results, it can be concluded that the addition of GEO to chitosan films opens up a promising future for biodegradable packaging applications with antimicrobial attributes for the packaging of meat, dairy, and horticultural products. 

## 4. Materials and Methods

### 4.1. Materials

The shrimp shells used in the study were procured from the local market in Basrah city, Iraq. Shrimp shells were washed with distilled water and dried at 50 °C for 12 h; they were then ground and stored in polyethylene bags at 4 °C until the required tests were performed. Acetic acid, sodium hydroxide, hydrochloric acid, and glycerol were obtained from Sigma (Munich, Germany). *Staphylococcus aureus* PTCC 1337, *Streptococcus* sp., *Bacillus subtilis*, *Salmonella* sp., *Pseudomonas aeruginosa*, and *Escherichia coli* 015:H7 were obtained from the Department of Food Science, University of Basrah, Basrah, Iraq. All strains were kept in nutrient broth (Sigma Aldrich, Munich, Germany) at −18 °C until further use.

### 4.2. Extraction of GEO

The essential oil extraction process was conducted in the laboratories of the Department of Food Science, College of Agriculture, University of Basrah. The ginger rhizomes (*Zingiber officinale*) were cleaned, washed and peeled, and finally EO was extracted by steam distillation for 3 h using a Clevenger-type apparatus. The EO was dried over anhydrous sodium sulfate and was later was stored in tightly closed containers at 4 °C prior to further usage.

#### GC-MS Characterization of GEO

The GEO was analyzed via GC/MS connected to a QP-2010 system and GC-2010 (Shimadzu Co., Kyoto, Japan). A capillary column DB-5 (0.25 mm × 0.25 mm id., film thickness 0.25 µm) was used to separate the GEO. The components of the EO were identified by comparing their mass spectra to the Wiley7n (Wiley, New York, NY, USA) library and NIST 08 lib mass spectral databases (National Institute of Standards and Technology, Gaithersburg, MD, USA). The peak area was used to calculate the relative percentages of the main components in the tested samples.

### 4.3. Chitosan Preparation

The chitin was prepared using the method mentioned by Trung et al. [79] with some modifications. The CH was prepared by three treatments, in which demineralization and deproteinization were the first steps. Namely, the shrimp shells (7.5 g) were treated with 1N HCl in the ratio of 1:4 (*w:v*) at room temperature for 30 min; then the shells were washed with distilled water four times to remove the mineral and calcium chloride salts. The next step was treatment of the shrimp shells with 1N NaOH 1:10 (*w:v*) at 65 °C for 1 h to remove proteins; then the solution was filtered through Whatman No.1 to eliminate the insoluble parts; the filtrate was washed with distilled water four times, then dried in an oven at 50 °C to obtain chitin. The preparation of CH involved deacetylation of the chitin [80]. Removing the acetyl groups from the chitin was achieved using 70% NaOH at 5 °C for 12 h. The CH was washed with distilled water more than once to eliminate the base, then it was poured into petri dishes and dried at 50 °C. The CH was ground into a fine powder and stored in light-permeable containers at 5 ± 2 °C before the next analysis.

### 4.4. Determination of Chemical Composition

Protein, moisture and ash contents of shrimp shells and chitosan were determined according to AOAC (2007) [81].

### 4.5. Antimicrobial Film Preparation

Bioactive films in this study were prepared by dissolving of 1 g of CH powder in a 100 mL (*w/v*) solution with 1% acetic acid, and 1% glycerol; stirred at 50 °C for 30 min with a magnetic stirrer until the system was homogenized. Afterward, the solution was cooled to room temperature, then 0, 0.1, 0.2, and 0.3% (*v/w*) of GEO was added and the solution was homogenized again for 30 min using a magnetic stirrer. The bioactive film solution was placed on petri dishes to dry at 50 °C overnight. The chitosan film without EO was utilized as a control. A final thickness of 4.5 ± 2 mm was determined in all samples. The dried films were peeled off and packed at 25 °C with 55 ± 2% RH for 48 h for further characterization [82].

### 4.6. Film Characterization

#### 4.6.1. Fourier-Transform Infrared Spectroscopy (FT-IR)

The effective (functional) groups were diagnosed according to Bonilla et al. (2014) [83]. The procedure was conducted in the following way: the film was mixed with potassium bromide KBr in a 100:1 ratio, then pressed under a pressure of 2500 kg/cm^2^ to obtain a tablet 1 mm in diameter and 1–2 mm thick. It was then measured by FT-IR (Jasco, Tokyo, Japan). FTIR spectra were recorded in the region between 4000 cm^−1^ to 400 cm^−1^.

#### 4.6.2. Differential Scanning Calorimeter (DSC) Analysis

Thermodynamic properties of films were determined using DSC-200F3 (Shimadzu, Tokyo, Japan) at a flow rate of 120 mL/min in a nitrogen atmosphere. The samples were prepared using a standard aluminum pan, and scanned over the temperature range of 30–400 °C with a heating rate of 20 °C/min. The curves of calorimetric analysis were recorded. The melting enthalpy (ΔHm), melting temperature (Tm), and thermal decomposition (Td) of the films were used to calculate the resulting thermoanalytical technique.

#### 4.6.3. X-ray Diffraction Analysis

X-ray diffraction (XRD) patterns were analyzed with an X per T-pro pw 3050 in ambient conditions using Cu- Kα radiation and a nickel monochromatic filtering wave at 40 kV and 20 mA. The biofilms were scanned from 2θ = 5–50 °C with a steep angle of 0.04 °C/min [83].

### 4.7. Antimicrobial Activity of BioActive Films

In vitro antimicrobial activity of chitosan-based films incorporating GEO was assessed by agar diffusion method, as per previous studies [25]. Inoculums of six spoilage and pathogenic bacteria, namely *Escherichia coli* 015:H7, *Staphylococcus aurous*, *Salmonella* sp., *Bacillus subtilis*, *Pseudomomus aeruginosa*, and *Streptococcus* sp., were prepared in Nutrient broth (Himedia, Mumbia, India) using an overnight culture of bacteria at 37 °C. The bioactive films, after sterilization with UV light, were cut into circular shapes of 6 mm in diameter and placed onto the Nutrient agar in Petri dishes. The Petri dishes had been previously inoculated with the inoculum (0.1 mL) by swabbing with approximately 10^4^–10^6^ CFU/mL of the tested bacteria. The diameter of the inhibition zone (mm) around the disc was measured after 24 h of incubation at 37 °C. Films without GEO were treated using the same protocol and were used as control.

### 4.8. Statistical Analysis

The results were analyzed statistically using a completely randomized design (CRD) with one factor and three replicates for each analysis. The significant differences between the averages were compared using the LSD test at the 0.05 level and using the (SPSS version 21.0 IBM Corp. Released 2012. IBM SPSS Statistics for Windows, Version 21.0., IBM Corp., Armonk, NY, USA).

## Figures and Tables

**Table 1:** Chemical composition of GEO using GC/GC-MS analysis.

Components	RT/min	%
α-Pinene	5.510	4.69
Camphene	5.925	15.07
α-Phellandrene	6494	0.23
β-Pinene	6.590	0.77
β-Myrcene	7.018	2.03
Eucalyptol	8.191	19.36
Norborneol	12.18	4.47
α-Terpineol	12.84	1.97
β-Bisabolene	15.03	11.52
Cineol	14.14	9.18
Lemomol	14.53	0.63
Borneol	15.23	0.33
α-Curcumene	20.29	1.25
α-Zingiberene	20.70	9.58
α-Farnesene	20.93	1.81
Terpinolene	20.97	1.21
γ-Elemene	21.20	0.71
β-Sequiphellandrene	21.35	3.31
Zingiberone	21.93	0.45
Nerolidol	22.26	0.29
Spathulenol	22.57	0.59
Globulol	22.74	0.48
Elemol	23.85	0.46
Β-Selinenol	24.49	1.42
Geraniol	25.25	0.57
Geranialdehyde	26.04	0.29
6-Gingerol	26.40	0.15

**Table 2:** Chemical composition of shrimp shell and CH.

Samples	Parameters (%)		
	Moisture	Ash	Protein
Shrimp shell	44.25 ± 2.21	31.40 ± 3.11	33.57 ± 2.87
Chitosan	6.89 ± 1.21	1.48 ± 1.92	3.72 ± 1.89

**Table 3:** DSC characteristics of examined samples based on CHf/GEO. (**a**) Chitosan; (**b**) Chitosan + 0.1% GEO; (**c**) Chitosan + 0.2% GEO; (**d**) Chitosan + 0.3% GEO.

Characteristics	Film Samples			
	CH (a)	CHf/GEO 0.1% (b)	CHf/GEO 0.2% (c)	CHf/GEO 0.3% (d)
∆H(J/g)	69.01	104.16	181.55	526.02
Tm	88.51	275.11	280.21	260.01
Ton	279.55	186.71	180.42	188.24
Tp	314.03	281.81	274.53	245.79

**Table 4:** Antimicrobial activity of bioactive films with GEO. Note: Different small letters indicate significant differences (*p* ≤ 0.05).

Bacterial Strains						
CHf with GEO %	*Escherichia coli* 015:H7	*Staphylococcus aureus*	*Salmonella* sp.	*Bacillus subtilis*	*Pseudomonas aeruginosa*	*Streptococcus* sp.
inhibition zone (mm)						
Control	8.81 ± 0.00 ^a^	11.44 ± 0.11 ^a^	9.77 ± 0.360 ^a^	12.80 ± 0.006 ^a^	10.72 ± 0.008 ^a^	11.38 ± 0.010 ^a^
0.1%	9.02 ± 0.01 ^b^	12.62 ± 0.00 ^b^	11.84 ± 0.017 ^b^	14.66 ± 0.005 ^b^	13.27 ± 0.008 ^b^	15.25 ± 0.00 ^b^
0.2%	11.38 ± 0.01 ^c^	14.91 ± 0.005 ^c^	12.57 ± 0.008 ^c^	15.26 ± 0.021 ^c^	13.36 ± 0.015 ^b^	15.44 ± 0.015 ^b^
0.3%	13.11 ± 0.02 ^d^	17.35 ± 0.01 ^d^	14.95± 0.017 ^d^	17.52 ± 0.008 ^d^	14.90 ± 0.012 ^c^	18.35 ± 0.012 ^c^

## Data Availability

Data available on request from the authors.
